# Draft Genome Sequence of *Mycolicibacterium* sp. Strain 018/SC-01/001, Isolated from the Marine Sponge *Iotrochota* sp.

**DOI:** 10.1128/MRA.01019-19

**Published:** 2019-10-17

**Authors:** Ji Fa Marshall Ong, Lik Tong Tan

**Affiliations:** a Natural Sciences and Science Education, National Institute of Education, Nanyang Technological University, Singapore, Singapore; University of Maryland School of Medicine

## Abstract

Here, we report the draft genome sequence of a marine bacterium, Mycolicibacterium sp. strain 018/SC-01/001, isolated from the marine sponge Iotrochota sp. collected from the Singapore Strait. The analysis of the bacterial genome using the bioinformatics tool antiSMASH 4.0.2 revealed the presence of a number of unique natural product biosynthetic pathways.

## ANNOUNCEMENT

There has been an increasing number of discoveries of structurally novel bioactive compounds with significant therapeutic activities that are produced by microbes isolated from marine samples, such as marine sediments and benthic invertebrates (1–5). Preliminary investigation carried out at our laboratory has revealed that there are potentially novel compounds produced by slow-growing marine bacterial strains associated with marine sponges collected in the Singapore Strait (4). Here, we have sequenced the genome of a representative *Mycolicibacterium* sp. 018/SC-01/001 strain isolated from samples of the marine sponge *Iotrochota* sp., collected using a rectangular dredge from the seabed surface in the Singapore Strait (1.155500°N, 103.735833°E) on 27 April 2017.

Homogenate from the sponge sample was processed in sterile artificial seawater, plated onto starch casein agar, and incubated at 25°C for 39 days before the marine bacterial colony was isolated and purified. Amplification of the 16S rRNA gene with the universal primers 27F (5′-GAGTTTGATCCTGGCTCAG-3′) and 1525R (5′-AGAAAGGAGGTGATCCAGCC-3′) and subsequent gene sequencing confirmed that isolate 018/SC-01/001 is a member of the Mycolicibacterium genus.

Genomic DNA (gDNA) was obtained from a culture of Mycolicibacterium sp. strain 018/SC-01/001 incubated at 25°C for 9 days in Difco marine agar. The gDNA was isolated using a Quick-DNA fungal/bacterial microprep kit (Zymo Research), followed by ethanol precipitation in order to obtain good-quality gDNA material prior to library preparation. Sequencing was performed using the 2 × 150-bp paired-end format on the MiSeq platform at Axil Scientific Pte. Ltd. (Singapore). The library was prepared using a Nextera XT library preparation kit (Illumina) following the manufacturer’s instructions. The library (22.8 nM) was sequenced using a 300-cycle MiSeq reagent v2 microkit (Illumina), yielding an average sequencing coverage of 40× and total reads of 3 Mb. The quality and quantity of the reads were determined using an Agilent TapeStation 4200 instrument, PicoGreen, and quantitative PCR (qPCR). Default parameters were used for all software unless otherwise specified. An initial annotation was made using the NCBI Prokaryotic Genome Annotation Pipeline using the best-placed reference protein set and GeneMarkS-2+ (6). The draft genome was found to be 5,781,079 bp in length, with a GC content of 68% and an *N*_50_ value of 198,717. A total of 49 contigs were obtained, with 48 containing protein-encoding genes, and there were 5,559 putative genes in total.

The sequence was examined using antiSMASH 4.0.2 (7, 8). A bacterial database was set as the reference depository, and gene cluster BLAST comparative analysis and the secondary metabolism gene family (secondary metabolism clusters of orthologous groups [smCOGs]) functions were selected with strict detection parameters that detect only well-defined clusters containing all required parts. A total of 16 biosynthetic gene clusters were identified and predicted, as follows: there are three clusters encoding the biosynthetic pathway for type 1 polyketide synthase (T1PKS), two type 1 polyketide synthase, nonribosomal peptide synthetase (T1PKS, NRPS) gene clusters, two type 3 polyketide synthase (T3PKS) gene clusters, two nonribosomal peptide synthetase (NRPS) gene clusters, three nonribosomal peptide synthetase-like (NRPS-like) gene clusters, two terpenes, one bacteriocin, and one arylpolyene cluster.

The *Mycolicibacterium* sp. 018/SC-01/001 genome sequence and the number of unique secondary metabolite biosynthetic gene clusters suggest that the bacterial strain may be a promising source of potentially novel molecules, as supported by the mass spectrometry-based metabolomics analysis of the organic extracts using the molecular networking platform (9).

### Data availability.

The raw sequence data have been deposited and made publicly available at DDBJ/ENA/GenBank under accession number PRJNA551549. The SRA data details have been deposited at DDBJ/ENA/GenBank under accession number SRX6373421. The whole-genome shotgun project has been deposited at DDBJ/ENA/GenBank under accession number VKAA00000000. The version described in this paper is the first version, VKAA01000000.
